# Iatrogenic Decompensated Heart Failure

**DOI:** 10.1007/s11897-020-00452-4

**Published:** 2020-02-21

**Authors:** Patrick Tran, Prithwish Banerjee

**Affiliations:** 1 Cardiology Department, University Hospital Coventry and Warwickshire, Clifford Bridge Road, Coventry, CV2 2DX UK; 2 Coventry University & Warwick Medical School, Coventry, UK

**Keywords:** Iatrogenic, Decompensated heart failure, Pharmacotherapy, Fluid management, High-output heart failure, Pacemaker

## Abstract

**Purpose of Review:**

To provide an overview of the potential iatrogenic causes of acute decompensated heart failure (AHF) and an evidence-based management strategy to address this.

**Recent Findings:**

As the heart failure (HF) population continues to age and become burdened with greater comorbidities and polypharmacy, patients become more susceptible to the iatrogenic precipitants of HF. The following clinical scenarios are familiar to clinicians, but the sequelae to AHF are often unanticipated: HF medications withdrawn during an intercurrent illness and not restarted, cardiotoxic therapy prescribed for cancer without timely and regular monitoring of left ventricular function, excessive intravenous fluids administered for sepsis or postoperatively, a blood transfusion volume not adjusted for body weight, iatrogenic anaemia that goes unnoticed or an inappropriate type of pacemaker implanted in a patient with underlying left ventricular systolic dysfunction.

**Summary:**

Iatrogenic decompensated HF is a phenomenon that is infrequently documented in the literature but increasingly confronted by clinicians of all specialties. It is associated with a high mortality and morbidity rate. By having greater awareness of these triggers, iatrogenic AHF should be one that is prevented rather than managed when it occurs.

## Introduction

One of the major challenges of managing acute decompensated heart failure (AHF) is identifying and addressing the precipitating factors, which are often multifactorial. The European Society of Cardiology (ESC) guidelines for heart failure (HF) emphasize on recognizing intrinsic cardiovascular triggers (such as acute coronary syndrome, arrhythmias and hypertension) and extrinsic insults such as infection and respiratory and renal dysfunction [1]. However, what is less described but seen increasingly more commonly in daily practice are precipitants related to inadvertent harm from acts of commission or omission by physicians, or directly by a form of medical therapy, which we collectively refer to as iatrogenic decompensated HF (IAHF). Little is known of its prevalence, and this type of data is not collected in the annual UK National Heart Failure Audit which analysed over 58,000 AHF hospitalisations [2]. An observational study in 1996 found that iatrogenesis accounted for 7% of HF admissions, and was associated with higher mortality and longer hospital stays compared with non-iatrogenic causes [3] though, this difference in mortality rate could have very likely been confounded by other comorbidities, additional medications or the presence of infection. With an aging population burdened with increasing comorbidities and polypharmacy combined with newer medications and technology, these seemingly innocuous therapies may unknowingly decompensate the delicate neurohormonal balance in these patients; hence, the current prevalence of IAHF is likely to be higher. An overview of these precipitants and its management implications is discussed under four major categories: pharmacotherapy, fluid management, high-output HF and pacemaker devices summarized in Table 1.

**Table 1:** Summary of potential iatrogenic causes for AHF

Pharmacotherapy	Withholding HF medications
	Delay in initiating HF medications
	Cardiotoxicity
	Adverse drug reactions
Fluid management	Excessive intravenous fluid Under-diuresis
	Transfusion-associated circulatory overload
	Dehydration
High-output HF	Arterio-venous fistula
	Anaemia
Pacemaker-related HF	Pacing-induced LV systolic dysfunction Pacemaker wire–related tricuspid regurgitation Pacemaker syndrome

## Pharmacotherapy

### Withholding and Delaying HF Medications

It is well-established that in patients with HF with reduced ejection fraction (HFrEF), renin-angiotensin-aldosterone system inhibitors (RAASi), e.g. ACE inhibitors (ACEi) and angiotensin-receptor blockers (ARBs), beta-blockers, mineralocorticoid-receptor antagonists (MRAs), the more recent combination sacubitril/valsartan, and sodium-glucose transport protein 2 inhibitors (regardless of diabetes status) markedly improve survival and reduce HF hospitalizations against placebo [4, 5]. The delay in starting, inappropriate discontinuation or failure to restart these prognostically vital medications can put these patients at risk of acute decompensation of stable chronic heart failure and sometimes cause haemodynamic deterioration.

RAASi is often misunderstood as a nephrotoxic drug. Introduction of the UK electronic acute kidney injury alert (AKI e-alert) system has exacerbated this anxiety, and a reflex cessation of RAASi amongst hospital and community practitioners occurs when a small serum urea or creatinine (sCr) rise is seen [6]. RAASi induces renal efferent arterial vasodilatation, and a resultant fall in intra-glomerular pressure is expected, reflected by an initial sCr rise and a decline in glomerular filtration rate (GFR) in the first 2 weeks. Moreover, GFR is dependent on blood pressure (BP). In HF patients who frequently have chronic kidney disease (CKD) and hypertension, the BP range for intra-renal autoregulation becomes narrower, so a small drop in BP can lead to a modest fall in GFR through RAASi-mediated vasodilation rather than intrinsic kidney injury [7]. New national guidance recommends withholding RAASi only if sCr increases by > 30% or potassium level ≥ 6.0 [8••]. It reminds physicians that an AKI e-alert does not automatically mean withdrawal of RAASi but instead, stimulate inquiry into other potential causes that may include AHF itself. AHF can lead to a disproportionate rise in urea via anti-diuretic hormone release, raised renal interstitial pressures in systemic venous congestion or functional ureteric obstruction from tense ascites [9, 10]. A study of > 16,000 patients found that discontinuation of RAASi in HFrEF patients is associated with higher mortality and re-admission rates at 30 and 90 days and 1 year [11]. In fact, RAASi offers the greatest mortality reduction in HFrEF patients with the worse renal function at baseline [12]. Therefore even with moderate-severe renal dysfunction, there is consensus to continue RAASi if benefits outweigh risks [8••].

The rationale follows for beta-blockers: the benefits are higher with increasing severity of HFrEF but with greatest risk when stopped. This is usually due to anxiety about its negative inotropic effects or in context of hypotension [13]. Unless there is severe hypotension, ESC recommends continuing beta-blockers at a reduced dose [1]. Besides, beta-blockers cannot be the reason for AHF in patients maintained on a long-term steady dose [14]. Discontinuation can trigger rebound tachycardia, ventricular arrhythmia and aggravate angina [15, 16]. This is associated with higher mortality and re-hospitalization rates [13]. Conversely, continuing beta-blockers in AHF correlates with lower mortality and admission rates [13, 17].

Even planned withdrawal of HF medications can lead to AHF in apparently asymptomatic chronic HF. In a prospective randomized trial of patients with stable dilated cardiomyopathy, MRA, beta-blockers and RAASi were sequentially weaned off. However, even with a controlled withdrawal of these medications, it resulted in a substantial fall in the left ventricular ejection fraction in 40% of patients, which was detected within 8 weeks [18]. This emphasizes the point that HF medications should only be temporarily withheld when absolutely necessary.

### Cardiotoxicity and Adverse Drug Reactions

Direct myocardial toxicity and fluctuations in afterload and preload are common mechanisms behind drugs implicated in AHF. An established phenomenon is cancer therapy–related cardiac dysfunction, whereby left ventricular ejection fraction (LVEF) falls by > 10 to < 53%, manifesting acutely or delayed, even as long as 20 years after completion of therapy [19]. Anthracyclines (e.g. doxorubicin) can cause dose-dependent cardiotoxicity that is generally irreversible, whereas biologically targeted drugs (e.g. trastuzumab for HER-2 positive breast cancer) can induce cardiotoxicity which is usually reversible upon prompt drug cessation or initiation of HF medications [20, 21]. According to ESC guidelines, a baseline echocardiogram is recommended before initiation of such therapy irrespective of the clinical history. Low-risk patients with a normal baseline echocardiogram and no clinical risk factors for HF should undergo echocardiography every 4 cycles of trastuzumab or after an equivalent dose of 200 mg/m^2^ anthracycline, while surveillance should be more frequent in high-risk patients. After completion of treatment, follow-up scans should be arranged at 1 and 5 years, and sometimes continued longer depending on the patient’s initial risk stratification. If clinical HF develops during or following treatment, it should be treated according to current ESC guidelines for HF with the initiation of cardio-protective medications such as ACEi and beta-blockers, usually prescribed together. Interruption or continuation of cancer treatment will depend on the risks versus benefits, determined by factors such as the degree of left ventricular systolic dysfunction (LVSD), cancer prognosis and responsiveness to the cancer therapy [1, 19, 22].

An estimated 50% of HF patients aged ≥ 65 suffer from at least 5 comorbidities. This results in greater exposure to other drugs such as steroids for chronic obstructive pulmonary disease, non-steroidal anti-inflammatory drugs (NSAIDs) for osteoarthritis and pioglitazone for type 2 diabetes mellitus (T2DM) [23]. These medications can increase fluid retention, afterload or preload, and potentially destabilize the neurohormonal and haemodynamics balance in HF. Steroids have a propensity to cause hypertension and fluid retention associated with a dose-dependent risk of precipitating or worsening HF [24]. A similar effect is seen in NSAIDs mediated by the inhibition of cyclo-oxygenase (COX) to trigger sodium and water retention. One study demonstrated a ten-fold increased risk of AHF over 72 months of NSAID use [25]. Indeed COX-2 inhibitors can also lead to a dose-dependent increased risk of HF hospitalization, and is discouraged in patients with ischaemic heart disease by ESC guidance [26, 27]. Fluid retention is also seen in alpha1-blockers (e.g. alfuzosin and doxazocin) and the oral hypoglycaemic agent, thiazolidinedione (e.g. pioglitazone). The former is associated with a two-fold risk of HF compared with other anti-hypertensives, while the latter is found to exacerbate existing HF and increase risk of de novo HF [28, 29]. Thus, these familiar drugs which are often prescribed in HF patients for other morbidities, e.g. osteoarthritis and T2DM, should be substituted for safer alternative.

Another comorbidity that frequently coexists is atrial fibrillation. For rate control, non-dihydropyridine calcium channel blockers should be avoided in HFrEF due to their negative inotropic activity and association with AHF [30]. For rhythm control, class I antiarrhythmics, e.g. flecainide, can significantly depress LV function and was found to precipitate AHF in patients with baseline LVSD in the CAST trial [31]. Another black-box warning is dronedarone (class III antiarrhythmic) for reasons alike and is contraindicated in patients with symptomatic HF [32].

Finally, AHF can occur postoperatively and one explanation may be the general anaesthesia. Inhalational agents, e.g. isoflurane and propofol, can lead to myocardial depression. Ketamine, a non-competitive NDMA glutamate receptor antagonist, exhibits both negative inotropy and sympathetic activation which may counteract the former. Nevertheless, in patients with significant baseline LV impairment, it can still decompensate HF [33].

## Fluid Management

### Excessive Intravenous Fluid and Under-Diuresis

The first Surviving Sepsis Campaign initiative recommended rapid intravenous fluid administration of up to 30 ml/kg to restore BP from third-space fluid losses [34]. However, this strategy is unsafe in patients with HF and sepsis. Achieving a mean arterial pressure (MAP) of 65 mmHg may not be realistic in some HFrEF patients who have lower than average baselines, and pushing more fluids to aim for 30 ml/kg can precipitate pulmonary oedema. This is supported by a retrospective study of patients with septic shock where intravenous fluid of 30 ml/kg as an early goal-directed therapy was associated with higher rates of fluid overload, which resulted in increased use of diuretics and thoracocentesis for pleural effusions [35]. This approach can even delay the anticipation and initiation of vasopressors or even mechanical circulatory support. The impact on survival between an initial liberal fluid strategy and restrictive fluids with early use of vasopressors in the setting of septic shock is still uncertain and of interest; CLOVERS is an ongoing multicentre randomized study investigating this question [36]. For now, international guidelines recommend an initial fluid challenge of 250-ml bolus over 30 min up to 500 ml with a low threshold to consider noradrenaline or dobutamine support on intensive care [37]. Similarly, judicious fluid management is imperative in surgical patients during the intraoperative and postoperative period. Apart from the obvious consequence of pulmonary oedema, liberal intravenous fluid is also associated with bowel oedema which can lead to ileus, poor intestinal absorption and bacterial translocation [38]. This can potentially delay the transition to oral hydration and delay the re-initiation and absorption of vital anti-heart failure medications, of which most are not available in intravenous form. Above all, the key is regular clinical assessment of fluid status.

Yet, fluid management will only be as accurate as the clinical evaluation of fluid status which can be challenging and contradictory between clinicians. Jugular venous pressure can be subjective and chest radiographic signs of HF can be non-specific [39, 40]. To complicate matters, elderly patients can present with subclinical congestion [41]. Hence, we frequently see the counterintuitive dilemma of treating with diuretics and intravenous fluids together. One study found 11% in-patients with AHF (without sepsis or bleeding) were given intravenous fluids (median 1000 ml) alongside diuretics, presumably to counterbalance any harm from over-diuresis. This increases LV filling pressures and prolongs the AHF episode [42]. Another fear to address is the initial rise in sCr with intravenous diuretics, swaying physicians to hold back on the furosemide dose, resulting in under-diuresis. The DOSE-AHF reassures us that high-dose intravenous furosemide versus low-dose regimen shows no differences in renal function over 60 days; rather, the former strategy offers greater diuresis without any significant renal impairment [43]. Patients with fluid overload should have diuretics up-titrated to achieve euvolaemia and not stopped or reduced prematurely based on renal function alone.

There is growing evidence that the use of N-terminal pro-B-type natriuretic peptide (BNP) and ultrasonography can help in fluid assessment. With serial BNPs, a falling level correlates well with a trend towards euvolaemia [44]. Echocardiography can reveal an inferior vena cava dilatation with little collapsibility to steer physicians away from further fluid administration, while lung ultrasound (LUS) can reveal bilateral diffuse vertical hyperechoic reverberations known as B-lines which can identify subclinical pulmonary oedema. The presence of ≥ 15 B-lines correlates with NT-proBNP > 1000 pg/ml and E/e′ ratio > 15 [45]. This may even lead to improved patient outcomes as demonstrated in a small randomized trial, LUS-HF, which found that recently discharged HF patients who were assessed using LUS during follow-up were associated with a significantly reduced rate of HF re-admissions [46]. Clinicians should thus integrate these newer technologies with physical examination for a more accurate judgement of fluid status.

### Transfusion-Related Circulatory Overload

Heart Failure, CKD and hypertension may be associated risk factors with transfusion-related circulatory overload (TACO) which manifests as pulmonary oedema within 6 h of transfusion and is associated with raised BP, BNP and a high mortality [47]. Small frail elderly adults are theoretically at greater risk of fluid overload if they receive a greater volume of blood transfusion than necessary. An average unit of packed red blood cells is 280 ml, and a dose of 4 ml/kg generally raises haemoglobin by 10 g/l [48]. Thus, an 80-year-old lady weighing 50 kg will only require 200 ml to raise haemoglobin by 10 g/l. However, if a whole unit is transfused and there is underlying HF, AHF may quickly follow. Although a Cochrane review indicated insufficient evidence, premedication with furosemide before blood transfusion remains common practice [49]. In contrast, volume depletion may precipitate AHF in the presence of LV outflow tract obstruction (LVOTO). A reduced LV volume and tachycardia can augment the intraventricular gradient and diastolic pressures precipitating pulmonary oedema. These are mainly reported through case series. For example, one case reported a patient with hypertrophic cardiomyopathy who developed significant LVOTO and pulmonary oedema after being kept nil by mouth and having received light sedation. This subsequently improved with intravenous fluids and beta-blockers [50]. Interestingly, mid-cavity LVOTO can also occur in up to 25% patients with Takotsubo cardiomyopathy predisposed by its basal myocardial hyperkinesia, excessive sympathetic stimulation and, in extreme cases, cardiogenic shock can ensue when inotropic agents are used [51, 52]. Overall, too much or too little intravenous fluids can trigger AHF if we overlook the clinical context.

## High-Output Heart Failure

### Anaemia

Severe anaemia can also worsen LVOTO as described above, but it is better known for unmasking myocardial ischaemia and causing high-output heart failure [53]. Anaemia can promote renal nitric oxide synthase activity. This causes peripheral vasodilation, neurohormonal activation, driving cardiac output (CO) above 8 l/min which over time leads to LV hypertrophy and dilation. All this can culminate to high-output HF [54, 55]. Anaemia is an independent predictor of HF mortality, yet a comorbidity that can go unnoticed [56]. It can be iatrogenic from surgical blood loss, post-catheterization haematuria or venepunctures, accelerated by anticoagulation or antiplatelets, and in turn, exacerbate a pre-existing anaemia of chronic disease from hepcidin upregulation [57]. Ferritin and transferrin saturation should therefore be routinely checked in HF. This can guide the prescription of intravenous iron which reduces HF hospitalization and improves exercise capacity, and is recommended over blood transfusion given the TACO risk [48, 58].

### Arterio-venous Fistula

Less commonly, the low systemic vascular resistance from a systemic arterio-venous shunt can similarly activate the systematic neurohormonal system. The degree of CO augmentation depends on the shunt size and magnitude of flow. Nephrologists should be wary that dialysis patients are at further risk of HF due to the additive effects of co-existing anaemia [55]. An arterio-venous fistula can be created inadvertently during vascular access e.g. in angiography or temporary pacing. A case report described a patient with AHF due to an iatrogenic femoral arterio-venous fistula from cannulation, and was found to have a CO > 16 l/min which was resolved after shunt closure [59]. Cardiologists should therefore be vigilant of this rare complication.

## Pacemaker-Related HF

A complication more familiar to cardiologists is pacing-induced cardiomyopathy defined by > 10% reduction in LVEF after pacemaker implantation (having excluded other causes). There are several mechanisms behind this [60]. Firstly, the pacemaker wire may affect closure of the tricuspid valve which may cause haemodynamically significant tricuspid regurgitation. This is uncommon. Secondly, with VVI pacing, if the sinus node discharges faster than the programmed rate, the atria will contract against a closed atrioventricular valve causing atrioventricular dyssynchrony (known as pacemaker syndrome) which can precipitate AHF. This can be relieved by either lowering the pacing rate or upgrading to a dual-chamber pacemaker (e.g. DDD) which supports a more physiological AV sequential pacing [61]. However, dual-chamber pacing can also precipitate AHF in patients with underlying HF. The DAVID trial found that in patients with underlying LVSD, DDD pacing (especially when right ventricular apical pacing (RVP) exceeded 40%) led to more HF hospitalization or deaths compared with ventricular back-up pacing. This is thought to be driven by electromechanical dyssynchrony and subsequent maladaptive LV remodelling. Indeed, when DDD resulted in < 40% RVP, the HF hospitalization rate was lower [62]. The BLOCK-HF trial shed light on this. In patients with high-grade AV block and LVEF ≤ 50%, biventricular pacing was associated with a 10% absolute risk reduction in death and AHF hospitalization compared with RVP [63]. As a result, those patients with HFrEF regardless of severity that require anti-bradycardia pacing are recommended to have biventricular pacing de novo or as an upgrade [1, 61].

## Conclusion

Iatrogenic decompensated HF is a phenomenon that is infrequently documented in the literature but increasingly confronted by clinicians of all specialties. It is associated with a high mortality and morbidity rate. As the HF population continues to age and become burdened with greater comorbidities and polypharmacy, patients become more susceptible to the iatrogenic precipitants of HF. Understandably, IAHF is sometimes unavoidable when concomitant renal dysfunction deteriorates, and the need to stop RAASi outweighs its benefit, or when an overarching concurrent illness develops, such as cancer which necessitates cardiotoxic chemotherapy. However, as long as clinicians are wary of the potential sequelae of AHF from these acts of commission or omission, we can anticipate and act promptly with timely monitoring of LV function and initiation of HF medications before AHF ensues. The already complex management of HF can be further complicated when a patient becomes acutely unwell with sepsis or significant anaemia. An effective yet simple approach would be regular clinical assessment integrated with the use of pulmonary ultrasound and biochemical markers such as BNP. Above all, by having greater awareness of these iatrogenic triggers, this phenomenon should be one that is prevented rather than managed when it occurs.
